# Characterization of Purple Acid Phosphatase Family and Functional Analysis of *GmPAP7a*/*7b* Involved in Extracellular ATP Utilization in Soybean

**DOI:** 10.3389/fpls.2020.00661

**Published:** 2020-06-24

**Authors:** Shengnan Zhu, Minhui Chen, Cuiyue Liang, Yingbin Xue, Shuling Lin, Jiang Tian

**Affiliations:** ^1^State Key Laboratory for Conservation and Utilization of Subtropical Agro-Bioresources, Root Biology Center, South China Agricultural University, Guangzhou, China; ^2^Department of Resources and Environmental Sciences, College of Chemistry and Environment, Guangdong Ocean University, Zhanjiang, China

**Keywords:** phosphorus deficiency, soybean, root-associated APase, purple acid phosphatase, ATP utilization

## Abstract

Low phosphate (Pi) availability limits crop growth and yield in acid soils. Although root-associated acid phosphatases (APases) play an important role in extracellular organic phosphorus (P) utilization, they remain poorly studied in soybean (*Glycine max*), an important legume crop. In this study, dynamic changes in intracellular (leaf and root) and root-associated APase activities were investigated under both Pi-sufficient and Pi-deficient conditions. Moreover, genome-wide identification of members of the *purple acid phosphatase* (*PAP*) family and their expression patterns in response to Pi starvation were analyzed in soybean. The functions of both *GmPAP7a* and *GmPAP7b*, whose expression is up regulated by Pi starvation, were subsequently characterized. Phosphate starvation resulted in significant increases in intracellular APase activities in the leaves after 4 days, and in root intracellular and associated APase activities after 1 day, but constant increases were observed only for root intracellular and associated APase activities during day 5–16 of P deficiency in soybean. Moreover, a total of 38 *GmPAP* members were identified in the soybean genome. The transcripts of 19 *GmPAP* members in the leaves and 17 in the roots were upregulated at 16 days of P deficiency despite the lack of a response for any *GmPAP* members to Pi starvation at 2 days. Pi starvation upregulated *GmPAP7a* and *GmPAP7b*, and they were subsequently selected for further analysis. Both GmPAP7a and GmPAP7b exhibited relatively high activities against adenosine triphosphate (ATP) *in vitro*. Furthermore, overexpressing *GmPAP7a* and *GmPAP7b* in soybean hairy roots significantly increased root-associated APase activities and thus facilitated extracellular ATP utilization. Taken together, these results suggest that GmPAP7a and GmPAP7b might contribute to root-associated APase activities, thus having a function in extracellular ATP utilization in soybean.

## Introduction

Phosphorus (P), an important macronutrient, is involved in many biochemical and metabolic processes in plants, such as photosynthesis, nucleotide synthesis, membrane remodeling, and protein modification (Liang et al., 2010; Zhang et al., 2014; Ham et al., 2018). However, a large proportion of P exists in immobile forms (i.e., organic P esters and inorganic complexes) and is thus unavailable for plant utilization in most soils, especially in acid soils (Chiou and Lin, 2011; Tian et al., 2012b; Plaxton and Lambers, 2015). Therefore, Pi fertilizer application is required to meet the Pi requirements for crop production. However, excess application of Pi fertilizer causes serious environmental eutrophication (Conley et al., 2009; Veneklaas et al., 2012; Abel, 2017; Wang and Liu, 2018). Thus, breeding cultivars with high P efficiency and optimizing field P management practices are necessary to maintain sustainable agricultural development (Tian et al., 2012b; Abel, 2017). It has been well documented that plants have evolved complex adaptation strategies to increase Pi foraging and recycling, such as altering root morphology and architecture, increasing organic acid and PAP exudation, and enhancing root–microbe interactions (Chiou and Lin, 2011; Liang et al., 2014; Ham et al., 2018; Jung et al., 2018).

In terms of adaptive strategies, plant PAPs are believed to play a vital role in Pi mobilization (Tran et al., 2010a; Plaxton and Lambers, 2015; Tian and Liao, 2015; Wang and Liu, 2018). It is generally observed that plant PAPs harbor a binuclear metal center binding either Fe (III)-Zn (II) or Fe (III)-Mn (II) ions and comprise five conserved motif blocks (DXG/GDXXY/GNH(D/E)/VXXH/GHXH) (Tran et al., 2010a; Tian and Liao, 2015). With the aid of genome sequence availability for plant species, a variety of PAPs have been identified and annotated. For example, there are 29 annotated PAP members in Arabidopsis (*Arabidopsis thaliana*), 26 in rice (*Oryza sativa*), 33 in maize (*Zea mays*), 25 in chickpea (*Cicer arietinum*), and 25 in physic nut (*Jatropha curcas*) (Li et al., 2002; Zhang et al., 2010; Gonzalez-Munoz et al., 2015; Bhadouria et al., 2017; Venkidasamy et al., 2019). Plant PAPs are widely divided into two major groups on the basis of PAP mass and structure: low molecular-mass monomeric PAPs, with a mass of approximately 35 kD, and high molecular-mass oligomeric PAPs, with a subunit mass of approximately 55 kD (Olczak et al., 2003; Tran et al., 2010a; Plaxton and Lambers, 2015; Tian and Liao, 2015).

It has been documented that P deficiency enhances the expression levels of most plant *PAPs*. For example, in rice, 10 out of 26 *PAP* members (*OsPAP1a*, *1d*, *3b*, *9b*, *10a*, *10c*, *20b*, *21b*, *23*, and *27a*) are upregulated in the roots under P-deficient conditions (Zhang et al., 2010). Similarly, Pi starvation enhanced transcripts of *PAP* members are also observed in 11 of 33 *PAP* members in maize, at least 20 of 25 *PAP* members in physic nut, 11 of 29 *PAP* members in Arabidopsis, 23 of 35 *PAP* members in soybean (*Glycine max*), and 12 of 25 *PAP* members in chickpea (Del Pozo et al., 1999; Haran et al., 2000; Li C. C. et al., 2012; Wang et al., 2014; Gonzalez-Munoz et al., 2015; Bhadouria et al., 2017; Venkidasamy et al., 2019). Therefore, it is suggested that increased transcription levels of *PAPs* could contribute to significant increases in intracellular and extracellular APase activities in plant species, such as Arabidopsis, wheat (*Triticum aestivum*), soybean, and tomato (*Lycopersicon esculentum*) (Del Pozo et al., 1999; Li et al., 2002; Kaida et al., 2003; Zimmermann et al., 2004; Liang et al., 2010; Chiou and Lin, 2011).

Recently, accumulating evidence has suggested that most PAP members participate in extracellular organic P utilization, including adenosine triphosphate (ATP), deoxynucleotide triphosphate (dNTPs), and phytate-P (Tran et al., 2010a; Plaxton and Lambers, 2015; Tian and Liao, 2015; Wang and Liu, 2018). A pioneer study on PAP function in extracellular ATP utilization has been conducted in common bean (*Phaseolus vulgaris*) in which overexpression of Pi starvation-enhanced *PvPAP3* resulted in significant increases of fresh weight and P content in bean hairy roots cultured in media supplemented with ATP as the sole P source (Liang et al., 2010). Similar functions have also been observed for other *PAP* members, such as *OsPAP10a*, *OsPAP21b*, *OsPAP26*, and *OsPAP10c* in rice (Tian et al., 2012a; Lu et al., 2016; Gao et al., 2017; Mehra et al., 2017). In addition to extracellular ATP utilization, plant PAP members have also been suggested to participate in extracellular dNTP utilization, including *PvPAP1*/*3* from common bean, *SgPAP7*/*10*/*26* from stylo (*Stylosanthes* spp.) and *GmPAP1-like* from soybean (Liang et al., 2012; Liu et al., 2016; Wu et al., 2018). Recently, some PAP members exhibiting phytase activity have been suggested to mediate extracellular phytate utilization, including *SgPAP23* from stylo, *OsPHY1* from rice, *MtPHY1* from Medicago (*Medicago truncatula*), and *GmPAP4* and *GmPAP14* from soybean (Ma et al., 2009; Li R. J. et al., 2012; Kong et al., 2014, 2018; Liu et al., 2018), suggesting diverse functions of *PAP* members in P scavenging and recycling in plants.

Soybean is an important legume crop species and an important oil and high-protein food or forage crop species worldwide (Conner et al., 2004; Herridge et al., 2008). In the face of low Pi availability conditions, soybean has evolved strategies to maintain Pi homeostasis, including the formation of a shallower root system, increased organic acid exudation and APase activities, and the formation of symbiotic associations with arbuscular mycorrhizal (AM) fungi (Tian et al., 2003; Zhao et al., 2004; Liao et al., 2006; Liu et al., 2008; Wang et al., 2010; Li et al., 2019). Furthermore, a functional analysis of several Pi starvation-responsive genes, such as *GmEXPB2*, *GmPHR25*, *GmPT5*/*7*, and *GmSPX1*/*3*, has highlighted molecular mechanisms underlying soybean adaptation to Pi starvation (Li et al., 2014, 2015; Yao Z. et al., 2014; Xue et al., 2017; Chen et al., 2018). However, the dynamic changes in intracellular and root-associated APase activities in soybean in response to Pi starvation, and the functions of GmPAP in extracellular ATP utilization remain unclear. Therefore, in this study, dynamic changes of intracellular (leaves and roots) and root-associated APase activities were investigated under both Pi-sufficient and Pi-deficient conditions. Moreover, genome-wide identification of members of the PAP family, and their expression patterns in response to Pi starvation were analyzed in soybean. Furthermore, the functions of the Pi starvation responsive *GmPAP7a* and *GmPAP7b* genes are suggested to participate in extracellular ATP utilization in soybean.

## Materials and Methods

### Plant Materials and Growth Conditions

The soybean genotype YC03-3 was used in this study. To analyze dynamic changes in APase activities at two P levels, soybean seeds were surface-sterilized and rolled in absorbent papers, which are soaked with one-fourth strength nutrient solution as described previously (Liang et al., 2010). After 5 days of germination, uniform seedlings were transferred to a nutrient solution comprising the following components (in μM): 1500 KNO_3_, 1200 Ca(NO_3_)_2_, 400 NH_4_NO_3_, 25 MgCl_2_, 500 MgSO_4_, 300 K_2_SO_4_, 0.3 (NH_4_)_2_SO_4_, 1.5 MnSO_4_, 0.5 CuSO_4_, 1.5 ZnSO_4_, 0.16 (NH_4_)_6_Mo_7_O_24_, 2.5 NaB_4_O_7_, 40 Fe-Na-EDTA, and 5 (−P) or 250 (+P) KH_2_PO_4_, as previously described (Wu et al., 2018). The nutrient solution was aerated hourly and refreshed weekly. Moreover, the pH value of the nutrient solution was adjusted to 5.8 every 2 days. The fresh weight of the soybean shoots and roots was determined daily within 7 days after P treatments were applied, and at 10, 13, and 16 days after P treatments were applied. Moreover, the roots were harvested to determine total P concentration, intracellular and root-associated APase activities. Primary leaves were also harvested to determine total P concentration and intracellular APase activities except at 0 and after 1 days of P treatment. To assay the temporal expression patterns of *GmPAP* members in response to Pi starvation, the primary leaves and roots after 2 days and 16 days of P treatments were also separately harvested for further analysis. All experiments had four biological replicates.

### Total P Concentration and APase Activity Analysis

The total P concentration was measured as described previously (Murphy and Riley, 1963; Xue et al., 2017; Mo et al., 2019). Briefly, approximately 0.1 g of dry samples was ground and digested using H_2_SO_4_-H_2_O_2_ reagent. Afterward, the mixtures were reacted with ammonium molybdate reagent and measured 30 min later at 700 nm.

To determine intracellular APase activities, the reaction product hydrolyzed by APase, ρ-nitrophenol (ρ-NP), was measured at an absorbance of 405 nm as described previously (Liang et al., 2010; Liu et al., 2016; Wu et al., 2018). Briefly, approximately 0.1 g of leaves and roots was ground and homogenized with 1.2 mL 0.1 M Tris–HCl (pH 8.0), and then the mixtures were centrifuged at 12,000 *g* for 30 min. After centrifugation, the supernatants were mixed with 1.8 mL of 45 mM Na-acetate buffer (pH 5.0) consisting of 1 mM ρ-nitrophenyl phosphate (ρ-NPP) as the substrate at 37°C for 15 min. After adding 1 M NaOH, the released ρ-NP was measured spectrophotometrically at 405 nm. The protein content in the supernatants was quantified using the Coomassie brilliant blue method (Bradford, 1976). Intracellular APase activities were expressed as the amount of ρ-NP produced per minute per milligram protein.

To analyze root-associated APase activities, seedlings grown in a nutrient solution containing 250 μM (+P) or 5 μM (−P) KH_2_PO_4_ were rinsed in a 0.2 M CaCl_2_ solution once and deionized water three times. After that, the whole roots of seedlings subjected to 0–7 days of P treatments and detached partial lateral roots of seedlings at 10–16 days of P treatment were transferred separately into tubes containing 40 mL of reaction buffer containing 45 mM Na-acetate buffer (pH 5.0) and 1 mM ρ-NPP. The reactions were incubated at 25°C for 2 h to allow the reactions to occur, and they were then terminated by the addition of 1 M NaOH. The absorbance of the reaction mixtures was separately measured at 405 nm (Liu et al., 2016). Moreover, the fresh weight of the roots was also determined. Root-associated APase activities were calculated as the amount of released ρ-NP per minute per gram of fresh roots.

To visualize root-associated APase activities, the roots were incubated in solid Murashige and Skoog (MS) media containing the substrate 5-bromo-4-chloro-3-indolyl-phosphate (BCIP) (Sigma-Aldrich, United States) as described previously (Wang et al., 2014). Briefly, the detached roots were placed on solid MS media containing 0.03% agar and then covered with solid MS media containing 0.06% agar and 0.1% (w/v) BCIP. After incubation at 28°C for 2 h, the cleavages of BCIP showed a bright blue color, which was imaged by a single-lens reflex camera (Canon, Japan). Each experiment was conducted for at least four biological replicates.

### Bioinformatic and Phylogenetic Tree Analysis of PAPs

For bioinformatic analysis, the sequences of PAP members from soybean were extracted from the NCBI^1^ and Phytozome^2^ databases. After multiple sequence alignment using ClustalX and conserved domain (metallophos domain) searches using the SMART tool^3^ (Bhadouria et al., 2017), GmPAP members were identified and named on the basis of their homology with corresponding PAP members in Arabidopsis. Detailed information about the GmPAP members, including their protein mass, number of amino acids, subcellular localization, and N-glycosylation site prediction, was retrieved separately from servers, including the ExPASy^4^, TargetP 1.1^5^ and NetNGlyc 1.0 server^6^ as described previously (Bhadouria et al., 2017; Xue et al., 2017; Yin et al., 2019). Moreover, a group of PAP members with known functions from other species, such as common bean, rice, maize, Arabidopsis, lupinus (*Lupinus luteus*), sweet potato (*Ipomoea batatas*), wheat, tobacco (*Nicotiana tabacum*), barely (*Hordeum vulgare*), stylo, Medicago, white lupin (*Lupinus albus*), potato (*Solanum tuberosum*), and chickpea, were retrieved from the NCBI database (Kaida et al., 2003; Li and Wang, 2003; Ma et al., 2009; Dionisio et al., 2011; Li C. C. et al., 2012; Madsen et al., 2013; Bhadouria et al., 2017; Liu et al., 2018). A phylogenetic tree was constructed using MEGA 5.01 with 1000 bootstrap values using the neighbor-joining method as described previously (Xue et al., 2017).

### RNA Extraction and Quantitative RT-PCR Analysis

Total RNA was extracted and purified as described previously (Xue et al., 2018). To eliminate genomic DNA contamination, the total RNA was further treated with RNase-free DNase I (Invitrogen, United States), and the purity was evaluated via A260/A280 ratios by using a Nanodrop spectrophotometer (Thermo, United States). Approximately 1 μg of RNA was reversely transcribed using MMLV-reverse transcriptase (Promega, United States) following the manufacturer’s protocols. Synthesized first-strand cDNA was used for SYBR Green-monitored quantitative RT-PCR (qRT-PCR) analysis on a Rotor-Gene 3,000 real-time PCR system (Corbett Research, Australia). The primers used for *GmPAP* members and the housekeeping gene *GmEF1-*α (*Glyma.17G186600*) are detailed in the Supplementary Table S1. Relative transcript levels of soybean *GmPAP* members were also presented using a heatmap generated by TBtools software.

### Subcellular Localization and Histochemical Localization of *GmPAP7a*/*7b*

The full-length *GmPAP7a*/*7b* coding sequence without a stop codon was separately amplified using the gene specific primers *GmPAP7a*/*7b-GFP-F*/*R* (Supplementary Table S1) and then cloned into a *pEGAD* vector to generate *35S:GmPAP7a*/*7b-GFP* fusion constructs. The constructs fusion with GFP and the plasma membrane marker *AtPIP2A*-mCherry were co-transformed into tobacco epidermal cells as described previously (Liu et al., 2016). The fluorescent signals were observed via a Zeiss LSM7 Duo confocal microscope (Zeiss, Germany) at 488 nm for GFP and 568 nm for mCherry (Liu et al., 2018). Fluorescent images were further processed using Zen2011 software (Carl Zeiss Microscopy, Germany). A 3.0 kb upstream sequence of *GmPAP7a*/*7b* from the start of codon was amplified using primers *GmPAP7a*/*7b-GUS-F*/*R* (Supplementary Table S1) and then inserted into a *pTF102* plasmid containing β*-glucuronidase* (*GUS*). The plasmids were subsequently transformed into soybean hairy roots for analysis of their histochemical localization. Transgenic soybean hairy roots were cultivated in MS media supplied with 1250 μM (+P) or 5 μM KH_2_PO_4_ (−P) for 1 week and then incubated in GUS staining solution (0.1 M Na_2_HPO_4_/NaH_2_PO_4_ buffer, pH 7.0, 1 mM X-Gluc) for 8 h at 37°C as described previously (Jefferson et al., 1987). After GUS staining, the root samples were separately observed under a light microscope (Leica, Germany).

### Purification and Biochemical Characterization of *GmPAP7a/7b*

The entire coding sequences of both *GmPAP7a* and *GmPAP7b* were separately cloned into a *pGEX-6P-3* vector (GE Healthcare, United States) constructs fusion to a GST tag using an in-fusion cloning kit (TAKARA, United States) with primers *GmPAP7a/7b-GST-F/R* (Supplementary Table S1). The expression constructs were introduced into *Escherichia coli* (*E. coli*) strain BL21. Recombinant proteins were extracted using BeaverBeads^TM^ GSH magnetic beads (Beaver Nano, China) and recognized by immunoblot analysis using anti-GST antibodies as described previously (Liu et al., 2018). Purified proteins were used to analyze their enzymatic properties. The effects of different pH values (ranging from 3.0 to 9.0) on GmPAP7a/7b activities were estimated separately by the addition of 5 mM ρ-NPP to the reaction buffers for 15 min at 37°C as described previously (Liu et al., 2018). The reaction buffer included 45 mM of glycine-HCl buffer (pH 3.0–4.5), Na-acetate buffer (pH 5.0–5.5), Tris–HCl 2-(N-morpholino)-ethanesulfonic acid (MES) buffer (pH 6.0–7.0), and Tris–HCl buffer (pH 7.5–9.0). Moreover, the effects of different temperatures (over a range of 20–80°C) on the enzymatic activities were also measured in 45 mM Na-acetate buffer (pH 5.0) with 5 mM ρ-NPP used as a substrate. The relative activities of GmPAP7a/7b were calculated as the percentages of their activities out of the highest activities under different pH values or temperatures. Moreover, a broad range of substrates were added to 45 mM Na-acetate buffer (pH 5.0) to test their substrate specificities, including the following (at concentrations of 5 mM): phytate-P, ATP, adenosine diphosphate (ADP), adenosine monophosphate (AMP), glucose-6-phosphate (G-6-P), glycerol-2-phosphate (G-2-P), guanosine monophosphate (GMP), inosine monophosphate (IMP), phospho-threonine (P-Thr), phospho-serine (P-Ser), and ρ-NPP. The relative activities were shown as the amount of Pi released from different substrates against the Pi released from ρ-NPP as the substrate. To determine the effects of different metal ions on GmPAP7a/7b activities, a total of nine different metal ions were applied at a final concentration of 5 mM: Mg^2+^, Fe^2+^, Al^3+^, Mn^2+^, Zn^2+^, Cu^2+^, Ba^2+^, Ca^2+^, and Co^2+^. The metal ions and GmPAP7a/7b proteins were separately incubated in reaction mixtures which consisted of 45 mM Na-acetate buffer (pH 5.0) and 5 mM ρ-NPP to analyze their activities. The relative activities were expressed as the activities with different metal ions divided by the activities without the addition of metal ions. Furthermore, the *V*_max_ and *K*_m_ values of GmPAP7a/7b toward ATP substrate were determined using a Lineweaver–Burk double reciprocal plot at different ATP concentrations. The units were expressed as the amount of Pi released per minute per milligram protein. All the data are shown as the means of three independent experiments.

### Functional Analysis of *GmPAP7a*/*7b* in Soybean Hairy Roots

The open reading frame of *GmPAP7a*/*7b* was separately amplified using specific primers (*GmPAP7a*/*7b*-*OX*-*F*/*R*) and subsequently cloned into a *pTF101s* binary vector. This enables glufosinate ammonium selection for positive transformants. *Agrobacterium rhizogenes*-mediated soybean hairy root transformation was performed as described previously (Wu et al., 2018). Briefly, YC03-3 seeds were surface sterilized and germinated on half-strength MS media for 4 days. The cotyledons were wounded and then transferred to MS media containing 100–200 μg L^–1^ glufosinate ammonium and 500 μg mL^–1^ carbenicillin disodium. After 14–20 days of growth, 18 independent transgenic lines, including nine overexpression lines and nine empty controls (verified by qRT-PCR), were selected for ATP utilization analysis as described previously (Liang et al., 2010). Briefly, approximately 0.1 g of transgenic soybean hairy roots was transferred to new solid MS media containing 0.4 mM ATP (Sigma-Aldrich, United States) or 6.25 μM KH_2_PO_4_ (−P), separately. After the root grew for 14 days, intercellular and root-associated APase activities, root fresh weight, and total P content were measured.

### Statistical Analyses

Data analyses and standard error calculations were statistically performed using Microsoft Excel 2016 (Microsoft Company, United States), and *t-*tests were performed with the SPSS program (v21.0; SPSS Institute, United States).

## Results

### Dynamic Effects of Pi Starvation on Soybean Biomass and P Concentration

**FIGURE 1:**
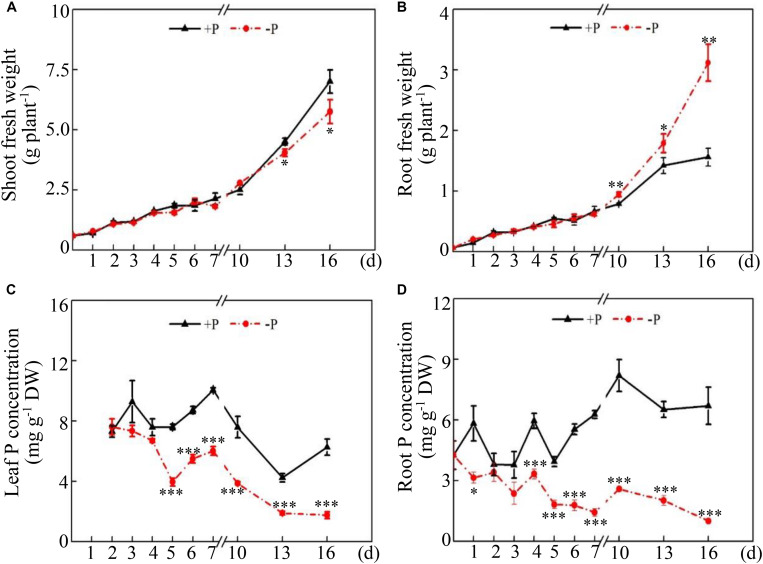
Dynamic changes in soybean growth and P concentration at two P levels. **(A)** Shoot fresh weight. **(B)** Root fresh weight. **(C)** Leaf P concentration. **(D)** Root P concentration. Soybean seedlings were grown in nutrient solution supplemented with 5 μM (–P) or 250 μM (+P) KH_2_PO_4_. Fresh weight and P concentration were dynamically measured. The data are the means of four replicates with standard errors. The asterisks indicate significant differences between the –P and +P treatments according to Student’s *t*-test: ^∗^*P* < 0.05; ^∗∗^ 0.001 < *P* < 0.01; ^∗∗∗^*P* < 0.001.

To investigate the effects of low Pi availability on soybean growth, the fresh weight of soybean shoots and roots and the total P concentration in both the leaves and roots were determined on different days after two P treatments. Although both shoot and root fresh weight increased during the period of the two P treatments, Pi availability exhibited different effects on dynamic changes in shoot and root fresh weight (Figures 1A,B). For shoot fresh weight, there was no difference between the +P and −P treatments during the early period of P treatments (i.e., 0–10 days), but shoot fresh weight under −P conditions was 10% and 17% less than that under +P conditions at 13 days and 16 days, respectively (Figure 1A), suggesting that Pi starvation significantly inhibited shoot growth after 13 days of P treatment. However, root fresh weight increased in response to Pi starvation at 10 days and reached the highest at 16 days, which was 99% more than that under +P conditions (Figure 1B), suggesting that Pi starvation enhanced soybean root growth. In contrast, significant decreases in total P concentration were observed in both the leaves and roots in response to Pi starvation, as reflected by the 48.1% decrease in the leaves at 5 days and the 46.1% decrease in the roots at 1 day (Figures 1C,D). Furthermore, the ratios of leaf P concentration at low P levels to that at high P levels were approximately 0.9 at 4 days and 0.3 at 16 days, but the ratios in the roots were 0.6 at 4 days and 0.2 at 16 days (Figures 1C,D). This suggests that Pi homeostasis in roots seems more susceptible to Pi availability than that in the leaves.

### Phosphate Starvation Increases APase Activities in the Leaves and Roots

**FIGURE 2:**
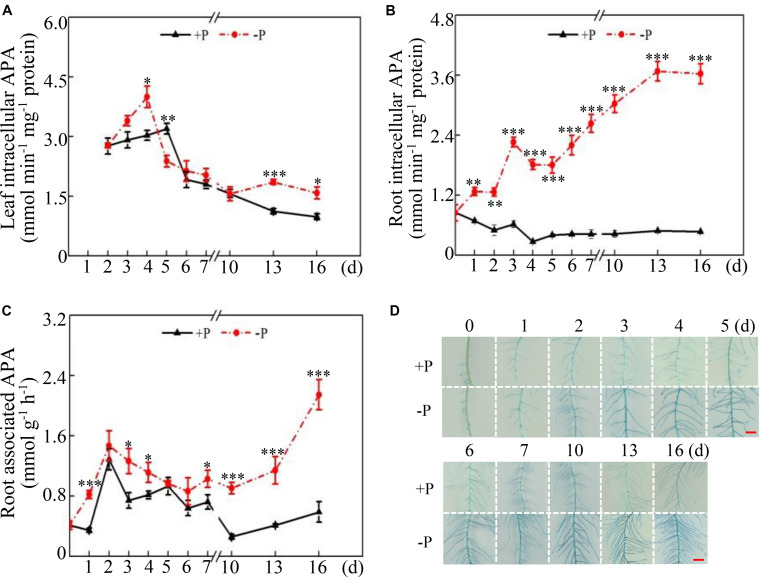
Dynamic changes in intracellular and root-associated APase activities in soybean. **(A)** Leaf intracellular APase activities. **(B)** Root intracellular APase activities. **(C)** Root-associated APase activities. **(D)** Root-associated APase activities detected by BCIP staining. Soybean seedlings were grown in nutrient solution supplemented with 5 μM (–P) or 250 μM (+P) KH_2_PO_4_. Intracellular and associated APase activities were dynamically measured. Data are means of four replicates with standard errors. Asterisks indicate significant differences between the –P and +P treatments by according to Student’s *t*-test: ^∗^*P* < 0.05; ^∗∗^ 0.001 < *P* < 0.01; ^∗∗∗^*P* < 0.001. The bars = 1 cm.

Dynamic changes in intracellular and root-associated APase activities were further investigated in both leaves and roots at two P levels. Distinct changes in APase activities were observed between leaves and roots under two P conditions. In the leaves, intracellular APase activities reached their highest levels at 4 and 5 days under low- and high-P conditions, respectively, followed by significant decreases with increased duration of P treatment (Figure 2A). Furthermore, leaf intracellular APase activities at low P levels were significantly higher than those at high P levels at 4, 13, and 16 days (Figure 2A), strongly suggesting that low Pi availability significantly affected intracellular APase activities in the leaves. Unlike changes in leaf intracellular APase activities, root intracellular APase activities were significantly enhanced at 1 day and increased by approximately 7-fold at 16 days in response to by Pi starvation (Figure 2B). Furthermore, root intracellular APase activities were gradually enhanced with increased duration of Pi starvation, but they remained unchanged after 5 days of high P treatment (Figure 2B). Although changes of root-associated APase activities exhibited a similar trend between the two P levels, Pi starvation led to more increases of root-associated APase activities after 10 days of P treatment (Figure 2C). Especially at 16 days, root-associated APase activities at low P levels were approximately 4-fold higher than those at high P levels (Figure 2C). When BCIP served as the substrate, root-associated APase activities were also visualized. A more intense blue color was detected on root surface under low-P conditions than that under high-P conditions, especially at 10, 13, and 16 days (Figure 2C), strongly suggesting that Pi starvation enhanced root-associated APase activities in soybean.

### Bioinformatic and Phylogenetic Analyses

When sequence homology alignment and conserved PAP domain analysis were used, compared to the 35 GmPAP members identified previously (Li C. C. et al., 2012), five new GmPAP members were identified, and two GmPAP members were not found in the updated soybean genome database. Therefore, a total of 38 GmPAP members were annotated and named in terms of sequence homology with corresponding AtPAP members by using multiple alignments and phylogenetic analysis. The general information of *GmPAP* members, including chromosomal location, length of coding sequence (CDS), and protein mass, is summarized in Table 1. The *GmPAP* members were found to be localized on different chromosomes (Table 1). The protein mass of the GmPAP family varied from 35 kD (GmPAP7d) to 74 kD (GmPAP9), and the full length of the CDS and number of amino acids varied from 933 to 1,989 bp, and from 310 to 662 aa, respectively (Table 1). Most GmPAP members were predicted to be involved in the secretory pathway. However, GmPAP22a and GmPAP22c were localized in the chloroplasts; GmPAP26b, GmPAP15b, and GmPAP27c in the mitochondria; and GmPAP1a, GmPAP12a, and GmPAP18b in other subcellular organelles (Table 1). Moreover, all GmPAP members except GmPAP27c and GmPAP7d were predicted to be modified by glycans (Table 1).

**TABLE 1:** General information of *GmPAP* members in soybean.

Name	Locus	Chromosomal location	Length of ORF (bp)	Number of amino acids (aa)	Protein size (kD)	Subcellular location	N-glycosylation sites
*GmPAP1a*	Glyma.02G117000.1	2	1926	642	72	–	5
*GmPAP18a*	Glyma.02G290100.1	2	1365	454	51	S	1
*GmPAP22a*	Glyma.03G194100.1	3	1476	491	55	C	2
*GmPAP20a*	Glyma.03G194200.1	3	1293	432	48	S	3
*GmPAP27c*	Glyma.04G195600.1	4	1560	519	59	M	0
*GmPAP27a*	Glyma.05G047900.1	5	1875	524	70	S	5
*GmPAP7d*	Glyma.05G138300.1	5	933	310	35	S	0
*GmPAP7a*	Glyma.05G138400.1	5	1008	335	37	S	2
*GmPAP17d*	Glyma.05G247800.1	5	1065	354	40	S	1
*GmPAP17a*	Glyma.05G247900.1	5	996	331	37	S	1
*GmPAP12a*	Glyma.06G028100.1	6	1135	444	51	–	5
*GmPAP12b*	Glyma.06G028200.1	6	1425	474	54	S	4
*GmPAP27b*	Glyma.06G170300.1	6	1872	623	70	S	5
*GmPAP17b*	Glyma.08G056400.1	8	987	328	37	S	1
*GmPAP7e*	Glyma.08G093300.1	8	969	322	36	S	2
*GmPAP7c*	Glyma.08G093500.1	8	999	332	38	S	1
*GmPAP7b*	Glyma.08G093600.1	8	1008	335	38	S	1
*GmPAP1b*	Glyma.08G291600.1	8	1856	616	69	S	3
*GmPAP18b*	Glyma.08G314800.1	8	1410	469	53	–	1
*GmPAP15a*	Glyma.08G351000.1	8	1644	547	62	S	4
*GmPAP27d*	Glyma.09G225000.1	9	1923	640	71	S	5
*GmPAP10a*	Glyma.09G229200.1	9.	1395	464	53	S	5
*GmPAP22b*	Glyma.10G071000.1	10	1266	421	48	S	3
*GmPAP15b*	Glyma.11G255700.1	11	1398	466	52	M	7
*GmPAP10b*	Glyma.12G007500.1	12	1395	464	53	S	5
*GmPAP27e*	Glyma.12G012000.1	12	1980	659	73	S	6
*GmPAP10c*	Glyma.12G221100.1	12	1410	469	54	S	4
*GmPAP26a*	Glyma.13G161900.1	13	1428	475	54	S	2
*GmPAP10d*	Glyma.13G280500.1	13	1416	471	54	S	4
*GmPAP18c*	Glyma.14G024700.1	14	1383	460	52	S	1
*GmPAP26b*	Glyma.17G109400.1	17	1539	512	59	M	4
*GmPAP15c*	Glyma.18G001300.1	18	1719	572	56	S	6
*GmPAP1c*	Glyma.18G132500.1	18	1851	616	69	S	3
*GmPAP23*	Glyma.19G026600.1	19	1764	587	65	S	5
*GmPAP22c*	Glyma.19G193900.1	19	1473	490	55	C	1
*GmPAP20b*	Glyma.19G194000.1	19	1290	429	48	S	1
*GmPAP17c*	Glyma.20G011900.1	20	984	327	37	S	3
*GmPAP9*	Glyma.20G026800.1	20	1989	662	74	S	6

*For predicted subcellular location, “S”, “C”, “M”, and “–” mean secretory, chloroplast, mitochondrial, and any other location, respectively.*

**FIGURE 3:**
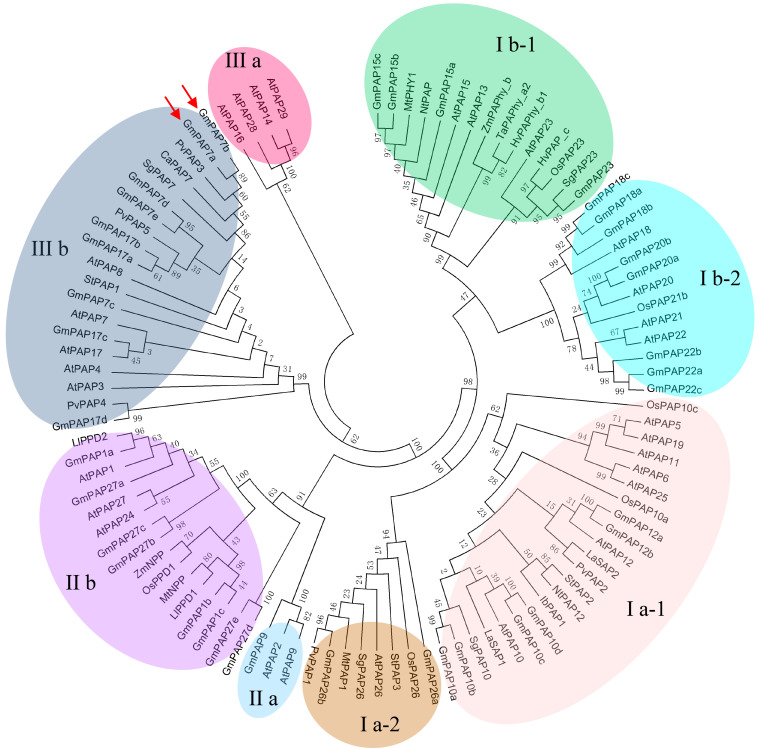
Phylogenetic analysis of PAPs in different plant species. The first two letters of each PAP indicate the abbreviated species name. At: *Arabidopsis thaliana*, Gm: *Glycine max*, Pv: *Phaseolus vulgaris*, Os: *Oryza sativa*, Zm: *Zea mays*, Ll: *Lupinus luteus*, Ta: *Triticum aestivum*, Nt: *Nicotiana tabacum*, Hv: *Hordeum vulgare*, Sg: *Stylosanthes guianensis*, Mt: *Medicago truncatula*, La: *Lupinus albus*, St: *Solanum tuberosum*, Ib: *Ipomoea batatas*, Ca: *Cicer arietinum*. The phylogenetic tree was constructed by MEGA 5.01 program using the neighbor-joining method, with 1000 bootstrap replicates. The bootstrap values are indicated for major branches as percentages. GmPAP7a and GmPAP7b are indicated by red arrows.

An unrooted phylogenetic tree was constructed by MEGA 5.01 using the neighbor-joining method; PAPs were idenRxied in various plant species, including common bean, rice, Arabidopsis, sweet potato, soybean, maize, lupinus, wheat, tobacco, barely, stylo, Medicago, white lupin, potato, and chickpea (Figure 3). It was observed that all plant PAPs could be mainly divided into three main groups comprising eight subgroups, all of which encompassed GmPAP members except subgroup III a (Figure 3). The molecular weights of the plant PAPs in group I and group II were approximately 55 kD and 75 kD, respectively, which were higher than those in the group III, of which the PAPs had a molecular weight of approximately 35 kD (Table 1 and Figure 3). Twenty out of 38 GmPAP members were assigned to group I. Notably, nine GmPAP members (GmPAP1a, 1b, 1c, 9, 27a, 27b, 27c, 27d, and 27e) were clustered into group II, and nine GmPAP members (GmPAP7a, 7b, 7c, 7d, 7e, 17a, 17b, 17c, and 17d) belonged to subgroup III b (Figure 3). Furthermore, GmPAP7a and GmPAP7b were close to PvPAP3 in common bean (Figure 3), which was reported to be involved in extracellular ATP utilization.

### Transcripts of *GmPAPs* in Response to Pi Starvation

**FIGURE 4:**
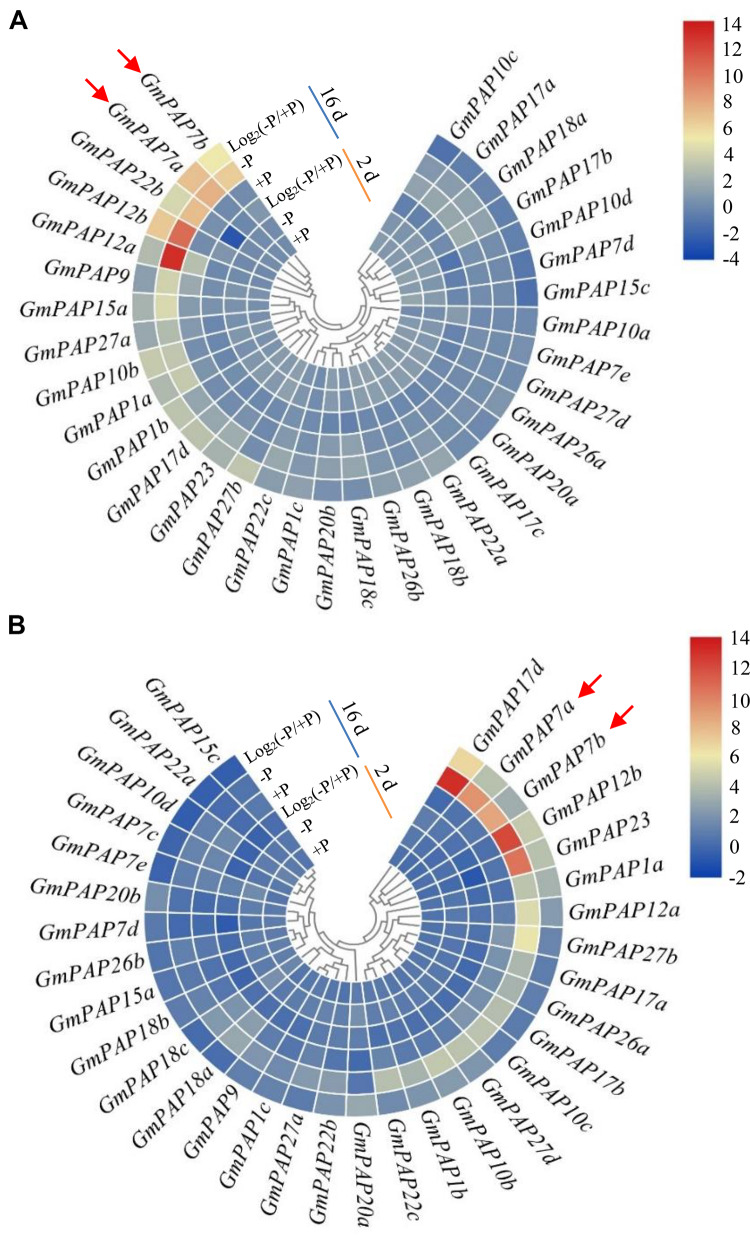
Heatmap analysis of the expression patterns of *GmPAP* members in leaves and roots in response to P deficiency. **(A)** Leaves. **(B)** Roots. Soybean seedlings were grown in nutrient solution supplemented with 5 μM (–P) or 250 μM (+P) KH_2_PO_4_. The leaves and roots were harvested for qRT-PCR analysis after 2 and 16 days of P treatment. Log_2_(–P/+P) represents the binary logarithm of the fold changes of the relative expression of *GmPAP* members under Pi-deficient and Pi-sufficient conditions. The data are the means of four replicates. *GmPAP7a* and *GmPAP7b* are indicated by red arrows.

Relative expression levels of *GmPAP* members in the leaves and roots were analyzed at 2 and 16 days at two P levels through qRT-PCR analysis. Transcripts of all *GmPAP* members were detectable at both P levels, except for those of four members in the leaves (i.e., *GmPAP27c*, *GmPAP27e*, *GmPAP7c*, and *GmPAP15b*) and for those of five in the roots (i.e., *GmPAP10a*, *GmPAP15b*, *GmPAP17c*, *GmPAP27c*, and *GmPAP27e*) (Figure 4 and Supplementary Table S2). At 2 days of P treatment, transcripts of the detected *GmPAP* members were not influenced by Pi availability in either the leaves or the roots (Figure 4 and Supplementary Table S2), suggesting that *GmPAP* members exhibited no response to early Pi starvation in soybean. However, at 16 days of P treatments, the transcript levels of nineteen genes (*GmPAP1a*, *1b*, *1c*, *7a*, *7b*, *9*, *10b*, *12a*, *12b*, *15a*, *17d*, *18b*, *22a*, *22b*, *22c*, *23*, *26b*, *27a*, and *27b*) were enhanced by more than 1-fold in the leaves of plants under Pi-deficient conditions compared with those under Pi-sufficient conditions (Figure 4A and Supplementary Table S2). Furthermore, five *GmPAP* members (*GmPAP7a*, *GmPAP7b*, *GmPAP12a*, *GmPAP12b*, and *GmPAP22b*) exhibited the highest expression levels in the leaves at low P level and clustered together (Figure 4A and Supplementary Table S2). In the roots, transcripts of 17 *GmPAP* members (*GmPAP1a*, *1b*, *1c*, *7a*, *7b*, *10b*, *10c*, *12a*, *12b*, *17b*, *17d*, *20a*, *22b*, *26a*, *23*, *27b*, and *27d*) were found to be significantly upregulated under Pi-deficient conditions (Figure 4B and Supplementary Table S2). Furthermore, five *GmPAP* members (*GmPAP7a*, *GmPAP7b*, *GmPAP12b*, *GmPAP17d*, and *GmPAP23*) exhibited the highest expression levels in the roots at low P level (Figure 4B and Supplementary Table S2). Overall, the expression levels of 12 *GmPAP* members (*GmPAP1a*, *1b*, *1c*, *7a*, *7b*, *10b*, *12a*, *12b*, *17d*, *22b*, *23*, and *27b*) were observed to be commonly increased in response to Pi starvation in both the leaves and roots, and this was especially the case for *GmPAP7a*, *GmPAP7b*, and *GmPAP12b* (Figure 4 and Supplementary Table S2).

### Tissue Expression Patterns and Subcellular Localization of *GmPAP7a/7b*

**FIGURE 5:**
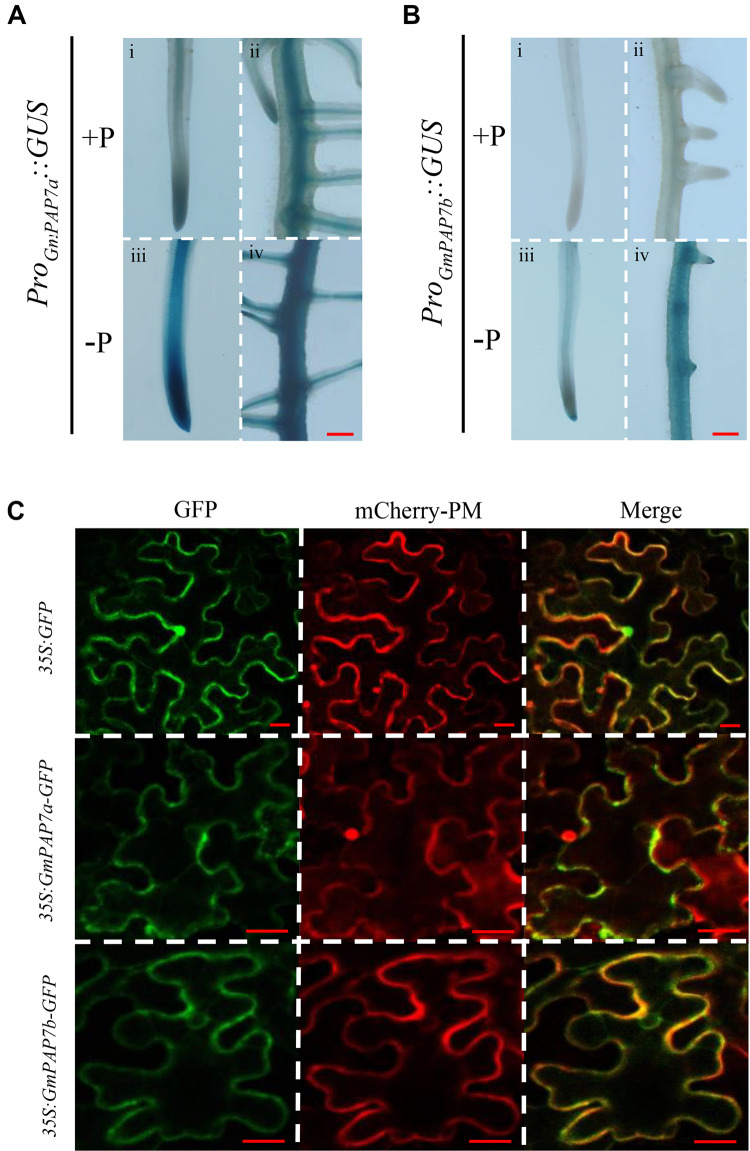
Histochemical and subcellular localization of *GmPAP7a*/*7b*. **(A)** GUS staining of transgenic soybean hairy roots harboring the *GmPAP7a* promoter (i–iv). **(B)** GUS staining of transgenic soybean hairy roots harboring the *GmPAP7b* promoter (i–iv). Transgenic soybean hairy roots were grown on MS media supplemented with 1250 (+P) μM or 5 μM (–P) KH_2_PO_4_ for 7 days, and then incubated with GUS staining for further analysis. Bars = 50 μm. **(C)** Subcellular localization of GmPAP7a and GmPAP7b. Transient expression of empty vector control (*35S:GFP*) or *35S:GmPAP7a*/*7b-GFP* constructs in tobacco leaf epidermal cells. Both GFP and mCherry-PM fluorescence were observed using confocal microscopy. mCherry-PM indicated the plasma membrane *AtPIP2A*-mCherry marker. Bars = 20 μm.

To further determine the tissue-specific expression patterns of *GmPAP7a*/*7b* in response to Pi starvation, *Pro_GmPAP__7__a_:GUS* and *Pro_GmPAP__7__b_:GUS* constructs were separately generated and introduced into soybean hairy roots subjected to P deficiency. Pi starvation led to higher GUS activity in both transgenic soybean hairy roots (Figures 5A,B), suggesting that Pi starvation could enhance *GmPAP7a*/*7b* transcript levels in the roots. Furthermore, under high P conditions, GUS activity was detected in both the root tips and steles of soybean hairy roots transformed with *Pro_GmPAP__7__a_:GUS* and only in the stele for *Pro_GmPAP__7__b_:GUS* (Figures 5A,B), suggesting that different tissue expression patterns might be present between *GmPAP7a* and *GmPAP7b* in roots under high P conditions. However, under low P conditions, GUS activity was separately detected in the whole roots transformed with two constructs (Figures 5A,B), suggesting that *GmPAP7a* and *GmPAP7b* exhibited similar expression patterns in roots at low P level.

To investigate their subcellular localization, *GmPAP7a* and *GmPAP7b* were separately fused to *GFP* and transiently over-expressed in tobacco leaves. The GFP signals of both *35S:GmPAP7a-GFP* and *35S:GmPAP7b-GFP* were detected mainly in the plasma membrane and cytoplasm (Figure 5C). However, the GFP signals of the control (*35S:GFP*) were detected throughout whole cells, including the plasma membrane, cytoplasm, and nucleus (Figure 5C). The results suggested that both GmPAP7a and GmPAP7b were located predominantly in the plasma membrane and cytoplasm (Figure 5C).

### Biochemical Characterization of *GmPAP7a/7b*

**FIGURE 6:**
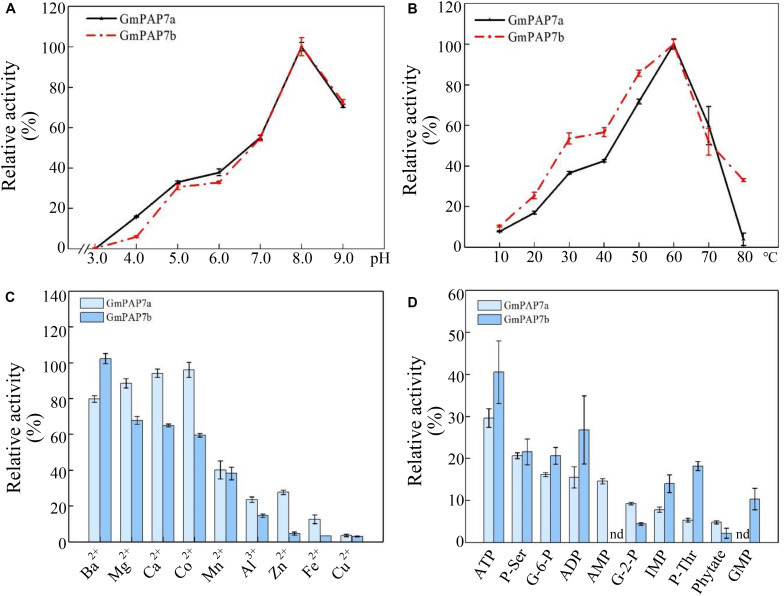
Enzymatic properties of GmPAP7a and GmPAP7b. **(A)** Effects of pH on the relative activities of GmPAP7a and GmPAP7b. **(B)** Effects of temperature on the relative activities of GmPAP7a and GmPAP7b. **(C)** Effects of metal ions on the relative activities of GmPAP7a and GmPAP7b. **(D)** Relative activities of GmPAP7a and GmPAP7b against different substrates. The data are the means of three replicates with standard errors. nd indicates that no activity was detected.

To determine the enzymatic properties of GmPAP7a/7b, recombinant proteins GmPAP7a-GST and GmPAP7b-GST were separately expressed and successfully purified from *E. coli* lysates (Supplementary Figure S1). The optimum pH and temperature for GmPAP7a/7b catalytic reactions were analyzed *in vitro* using ρ-NPP as the substrate, and it was observed that both GmPAP7a and GmPAP7b exhibited similar properties for optimum pH and temperature, as reflected by the maximum relative activities that were separately observed at pH 8.0 and 60°C (Figures 6A,B). However, GmPAP7a activities seem more sensitive to high temperature than GmPAP7b because the relative activities of GmPAP7a and GmPAP7b were 2% and 30% at 80°C, respectively (Figure 6B). In addition, the effects of metal ions on the activities of GmPAP7a and GmPAP7b were separately investigated (Figure 6C). The relative activities of both GmPAP7a and GmPAP7b were reduced by more than 50% by application of Mn^2+^, Al^3+^, Zn^2+^, Fe^2+^, and Cu^2+^, especially Cu^2+^, which inhibited their relative activities by 90% (Figure 6C). However, significant inhibition of relative activities was observed only for GmPAP7b and not for GmPAP7a, when Mg^2+^, Co^2+^ or Ca^2+^ was applied (Figure 6C).

**FIGURE 7:**
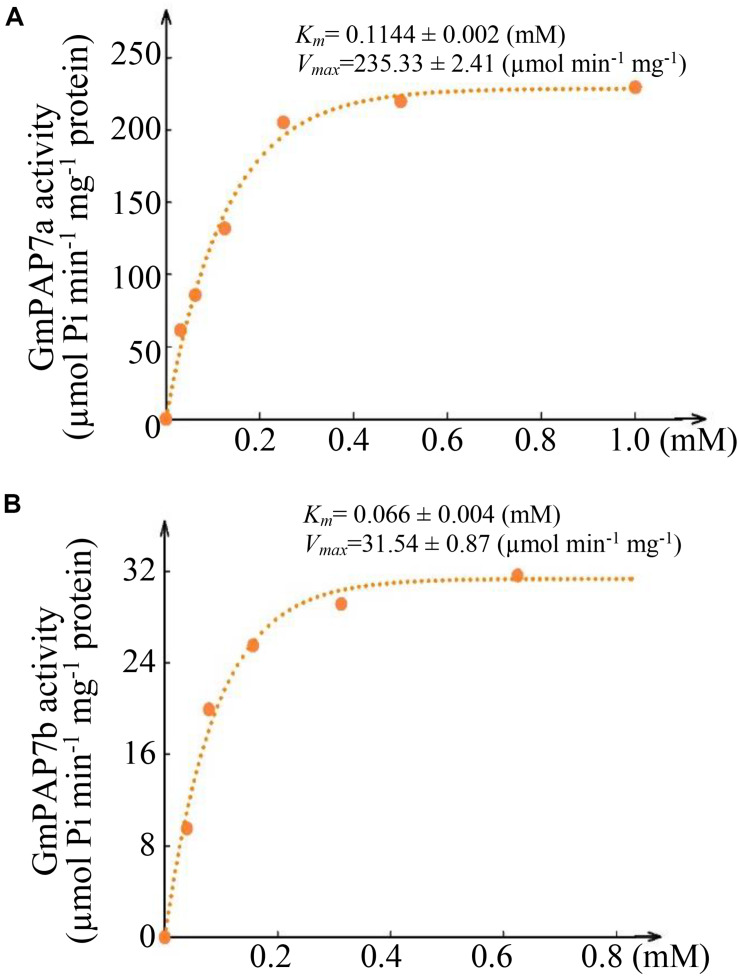
Kinetic parameters of GmPAP7a and GmPAP7b against ATP. **(A)** Kinetic parameter of GmPAP7a. **(B)** Kinetic parameter of GmPAP7b. The *K*_m_ and *V*_max_ of GmPAP7a and GmPAP7b were calculated from Lineweaver–Burke plots of enzyme activities at different concentrations of ATP. The data are the means of three replicates.

In a test of substrate specificity, the relative activities of both GmPAP7a and GmPAP7b were highest against ATP, followed by phosphate-serine for GmPAP7a and ADP for GmPAP7b (Figure 6D). Furthermore, activities against AMP were observed only for GmPAP7a, not for GmPAP7b. Similarly, activities against GMP were observed only for GmPAP7b and not for GmPAP7a (Figure 6D). When ATP served as the substrate, kinetic analyses showed that *K*_m_ and *V*_max_ values were 0.11 mM and 235.3 μmol min^–1^ mg^–1^ protein for GmPAP7a and 0.066 mM and 31.5 μmol min^–1^ mg^–1^ protein for GmPAP7b, respectively (Figure 7), suggesting that the catalytic properties differ between GmPAP7a and GmPAP7b.

### Overexpressing *GmPAP7a*/*7b* Enhanced Exogenous ATP Utilization

**FIGURE 8:**
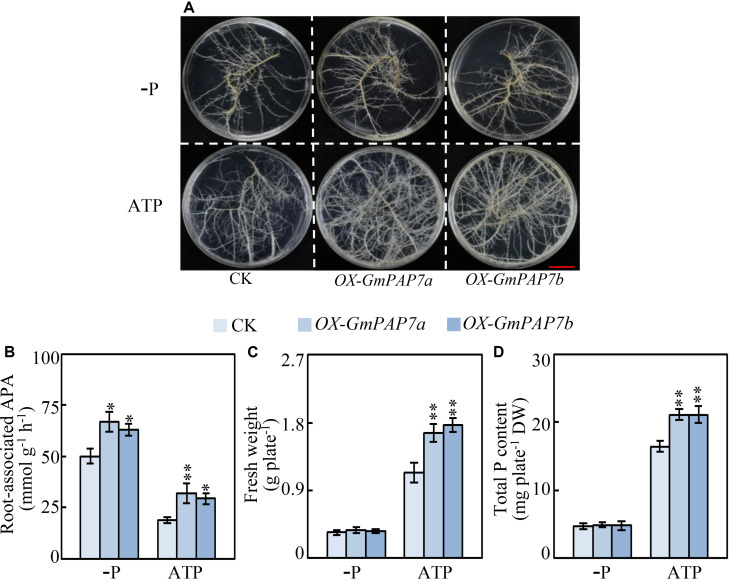
Effects of *GmPAP7a* and *GmPAP7b* overexpression on APase activities, total P content and fresh weight of transgenic soybean hairy roots. **(A)** Image of transgenic hairy roots supplied with different P sources. Bars = 1 cm. **(B)** Root-associated APase activities. **(C)** Fresh weight of transgenic soybean hairy roots. **(D)** Total P content in transgenic soybean hairy roots. Transgenic soybean hairy roots were grown for 14 days on MS media supplemented with 6.25 μM KH_2_PO_4_ (–P) or 0.4 mM ATP as the sole P source. Fresh weight and total P content were separately measured. CK represents transgenic hairy root lines transformed with an empty vector. *OX-GmPAP7a* and *OX-GmPAP7b* indicate transgenic hairy root lines overexpressing *GmPAP7a* and *GmPAP7b*, respectively. The data are the means of nine replicates with standard errors. The asterisks indicate significant differences between the overexpression and CK lines according to Student’s *t*-test: ^∗^*P* < 0.05, ^∗∗^*P* < 0.01.

To investigate the functions of *GmPAP7a* and *GmPAP7b* in exogenous ATP utilization, transgenic soybean hairy roots overexpressing *GmPAP7a* or *GmPAP7b* were generated (Figure 8). The increased expression levels of *GmPAP7a* or *GmPAP7b* in transgenic hairy root lines (*OX-GmPAP7a*/*7b*) were verified through qRT-PCR analysis, as reflected by more than 11-fold increases compared to those of the control (CK) lines (Supplementary Figure S2a). Moreover, overexpression of *GmPAP7a* and *GmPAP7b* led to significant increases in intracellular APase activities (Supplementary Figure S2b) and root-associated APase activities under P-deficient conditions and ATP application (Figure 8). Furthermore, the fresh weight and the total P content were approximately 50% and 28% higher, respectively, in the soybean hairy root lines with *GmPAP7a* or *GmPAP7b* overexpression than in the control lines with ATP application, while no difference was observed in the −P treatments (Figure 8), strongly suggesting that GmPAP7a and GmPAP7b might participate in extracellular ATP utilization in soybean.

## Discussion

Phosphorus is an essential macronutrient participating in many biochemical and metabolic processes (Liang et al., 2010; Zhang et al., 2014; Ham et al., 2018). Low Pi availability imposes serious limitations on crop growth and production (Vance et al., 2003; Zhang et al., 2014; Dissanayaka et al., 2018). It is generally observed that P deficiency alters root system to increase the root surface area and exploitable soil volume for Pi uptake (Pérez-Torres et al., 2008; Lambers et al., 2011; Lynch, 2011). In this study, enhanced soybean root growth was also observed in soybean in response to Pi starvation, as reflected by increased soybean root fresh weight after 10 days of Pi starvation (Figure 1), strongly suggesting that significant changes in the soybean root system occur under P-deficient conditions.

In addition to changes in root morphology and architecture, Pi starvation also results in increases in intracellular and extracellular APase activities in plants (Zimmermann et al., 2004; Plaxton and Lambers, 2015; Tian and Liao, 2015; Wang and Liu, 2018). Furthermore, accumulating evidence has suggested that increases in APase activities are mainly due to an enhanced *PAP* transcripts under low P conditions in plants, such as *PvPAP3* in bean, *AtPAP10*/*12* in Arabidopsis, *OsPAP10a* and *OsPAP21b* in rice (Liang et al., 2010; Tran et al., 2010b; Wang et al., 2011; Tian et al., 2012a; Mehra et al., 2017). In this study, root intracellular and associated APase activities were significantly enhanced by Pi starvation at 16 days (Figure 2), accompanied by significantly increased transcripts of 17 *GmPAP* members in the roots (Figure 4B and Supplementary Table S2), strongly suggesting that increased transcripts of *GmPAP* members could lead to enhanced APase activities in soybean. However, increases of root intracellular APase activities were also detected after 2 days of Pi starvation, while the transcript abundance of no *GmPAP* member changed in response to Pi starvation in either the leaves or roots (Figures 2, 4), suggesting that there must be other molecular mechanisms underlying APase activity regulation in addition to controlling the transcript levels of *GmPAP* members. In soybean, 36 out of 38 GmPAP members were predicted to exhibit more than one glycosylation site (Table 1). Moreover, it has been suggested that the enzymatic properties of plant PAPs could be impacted by their glycosylation (Navazio et al., 2002; Olczak and Olczak, 2007; Tran et al., 2010b). Therefore, it is plausible that glycosylation modification of GmPAP members may play a role in regulating APase activities in response to 2 days of Pi starvation, which merits further analysis.

With the aid of genome sequence availability in various plant species, increased transcripts of *PAP* members in response to Pi starvation have been found in plant species, such as Arabidopsis, rice, maize, and chickpea (Zhang et al., 2010; Wang et al., 2014; Gonzalez-Munoz et al., 2015; Bhadouria et al., 2017). Similarly, the expression levels of 19 and 17 *GmPAP* members increased in response to Pi starvation in the leaves and roots, respectively (Figure 4 and Supplementary Table S2), suggesting that increased *PAP* transcription is a common response to Pi starvation in plants. Moreover, a group of plant *PAP* members have been suggested to be downstream genes of *PHR1* (or its homologs), which is the central regulator in the P signaling network; these *PAPs* include *AtPAP17* in Arabidopsis, 10 *OsPAP* members in rice, and *PvPAP3* in bean (Rubio et al., 2001; Bari et al., 2006; Zhang et al., 2010; Yao Z. F. et al., 2014). These findings suggest that *GmPAP* members might also be regulated by *PHR1* or its homologs in soybean. Consistently, *GmPAP14* (i.e., *GmPAP7e*) and *GmPAP21* (i.e., *GmPAP22b*) have been suggested to be downstream genes of *GmPHR25* because *GmPHR25* overexpression leads to increased transcript levels in roots of *GmPHR25* overexpression composite plants (Xue et al., 2017). However, the regulatory mechanisms of other Pi starvation-responsive *GmPAP* members remain unclear.

Root-associated PAPs are well known to play a role in the utilization of extracellular organic P sources, such as ATP, phytate-P and dNTPs (Liang et al., 2010, 2012; Wang et al., 2011, 2014; Robinson et al., 2012; Liu et al., 2016, 2018; Lu et al., 2016; Gao et al., 2017; Mehra et al., 2017; Wu et al., 2018). For example, AtPAP10/12/26 are suggested to participate in the utilization of extracellular DNA and ADP in Arabidopsis (Wang et al., 2011, 2014). However, the functions of PvPAP3 in bean and OsPAP10a/21b/26/10c in rice are suggested to mediate extracellular ATP utilization (Liang et al., 2010; Tian et al., 2012a; Lu et al., 2016; Gao et al., 2017; Mehra et al., 2017). In this study, phylogenetic tree analysis revealed that PvPAP3, GmPAP7a, and GmPAP7b belonged to group III b (Figure 3), suggesting they might have similar functions in plants. Moreover, both GmPAP7a and GmPAP7b exhibited relatively high activities against ATP *in vitro* (Figure 6D) and localized to the plasma membrane and cytoplasm (Figure 5C), which was similar to that occurred for PvPAP3 (Liang et al., 2010). Furthermore, the fresh weight and total P content in soybean hairy roots with *GmPAP7a* and *GmPAP7b* overexpression were significantly higher than those of control lines with ATP application (Figure 8), strongly suggesting that GmPAP7a and GmPAP7b could utilize extracellular ATP. Interestingly, pH optima of GmPAP7a and GmPAP7b activity was 8.0 *in vitro* (Figure 6A). Similarly, pH optima of several plant PAP activity was found to be above 7.0 despite how the pH optima of activity for most plant PAPs is generally below 7.0 (Tran et al., 2010a; Plaxton and Lambers, 2015; Tian and Liao, 2015; Wang and Liu, 2018). For example, pH optima of PvPAP3 against ρ-NPP and KbPAP against ATP was 7.0 or above (Cashikar et al., 1997; Liang et al., 2010). Therefore, these results suggest that some plant PAPs might have functions as an alkaline phosphatase, which merits further study.

Remobilization of internal phosphorylated metabolites (e.g., phosphorylated carbon) is generally considered to be an adaptive strategy plants in responses to Pi starvation (Chiou and Lin, 2011; Tian et al., 2012b; Plaxton and Lambers, 2015; Abel, 2017). Consistently, decreased accumulation of phosphorylated compounds was widely observed in plants under Pi-deficient conditions (Chiou and Lin, 2011; Tian et al., 2012b; Plaxton and Lambers, 2015; Mo et al., 2019). Given that plant PAPs exhibit activity against a set of phosphorylated metabolites, it is suggested that internal plant PAP might play a role in metabolic processes of internal phosphorylated metabolites (Tian et al., 2012b; Plaxton and Lambers, 2015). In this study, since transcript levels of both *GmPAP7a* and *GmPAP7b* were also significantly upregulated in leaves after 16 d of P deficiency, it is plausible that GmPAP7a and GmPAP7b might participate in the recycling of internal phosphorylated metabolites in leaves.

In summary, a total of 38 GmPAP members were idenRxied in the soybean genome. However, the transcript levels of 19 and 17 *GmPAP* members were upregulated by 16 days of P deficiency in the leaves and roots, respectively. Among them, both GmPAP7a and GmPAP7b had the highest activities against ATP *in vitro*. Furthermore, overexpressing *GmPAP7a* and *GmPAP7b* resulted in significant increases in root-associated APase activities and total P content in soybean hairy roots when ATP was supplied as the sole P source. Taken together, these results suggest that Pi starvation responsive *GmPAP7a* and *GmPAP7b* may mediate root-associated APase activities and thus control extracellular ATP utilization in soybean in response to P deficiency.

## Data Availability Statement

All datasets generated for this study are included in the article/Supplementary Material.

## Author Contributions

JT and CL conceived and designed the experiments. SZ, MC, and SL performed the experiments. JT, CL, SZ, MC, and YX analyzed the data. JT, CL, SZ, and YX wrote the manuscript. All authors have read and approved the final manuscript.

## Conflict of Interest

The authors declare that the research was conducted in the absence of any commercial or financial relationships that could be construed as a potential conflict of interest.

## Footnotes

1 https://www.ncbi.nlm.nih.gov/pubmed

2 https://phytozome.jgi.doe.gov/pz/portal.html

3 http://smart.embl-heidelberg.de/smart/set_mode.cgi?NORMAL=1

4 https://web.expasy.org/protparam/

5 http://www.cbs.dtu.dk/services/TargetP-1.1/index.php

6 http://www.cbs.dtu.dk/services/NetNGlyc/

## Supplementary Material

FIGURE S1 Analysis of the expression and purification of recombinant GmPAP7a-GST and GmPAP7b-GST proteins.

FIGURE S2 Analysis of *GmPAP7a*/*7b* transcription and intracellular APase activities in transgenic overexpression soybean hairy roots.

TABLE S1 Primers used for qRT-PCR or vector construction.

TABLE S2 Relative expression levels of *GmPAP* members.

## Abbreviations

APase: acid phosphatase
P: phosphorus
PAP: purple acid phosphatase
Pi: phosphate
qRT-PCR: quantitative real-time polymerase chain reaction.
