# Supplementary material for: Characterization of Purple Acid Phosphatase Family and Functional Analysis of GmPAP7a/7b Involved in Extracellular ATP Utilization in Soybean
Source: Front Plant Sci. 2020 Jun 24;11:661. doi: 10.3389/fpls.2020.00661 (PMC7326820; doi:10.3389/fpls.2020.00661)
Supplement: FIGURE S1 — Analysis of the expression and purification of recombinant GmPAP7a-GST and GmPAP7b-GST proteins. [file Data_Sheet_1.PDF]

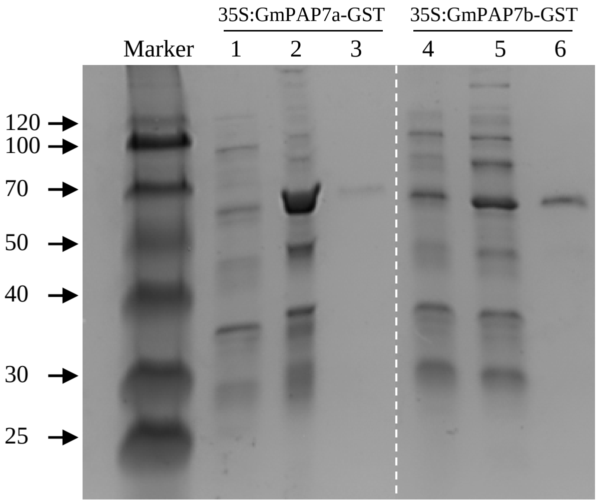

FIGURE S1. Analysis of the expression and purification of recombinant GmPAP7a-GST and GmPAP7b-GST proteins. Lane 1/4, total soluble crude protein extracted from *E.coli* transformed with *35S:GmPAP7a-GST* and *35S:GmPAP7b-GST*, respectively; Lane 2/5, precipitation of crude protein extracted from *E.coli* transformed with *35S:GmPAP7a* and *35S:GmPAP7b-GST*, respectively; Lane 3/6, purified GmPAP7a-GST and GmPAP7b-GST using BeaverBeads™ GSH magnetic beads.

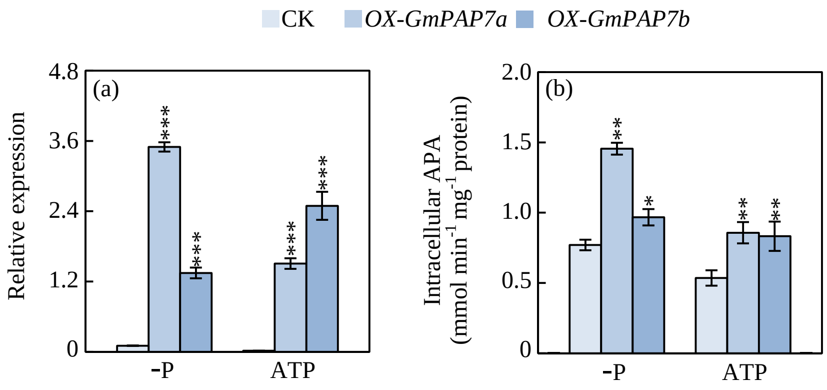

FIGURE S2. Analysis of GmPAP7a/7b transcription and intracellular APase activities in transgenic overexpression soybean hairy roots. (a) qRT-PCR analysis of the *GmPAP7a/7b* transcripts in soybean transgenic hairy roots with -P and ATP treatments. (b) Internal APase activities. CK represents the transgenic hairy root lines transformed with the empty vector. OX indicates the transgenic hairy root lines with overexpressing *GmPAP7a* or *GmPAP7b*. Transgenic hairy roots were grown on MS medium supplied with 6.25 μM KH_2PO_4 (-P), 0.4 mM ATP as the sole P source for 14 d. Data are means of nine replicates with standard errors. Asterisks indicate significant difference between overexpression and CK lines by Student's *t*-test: * $P<0.05$; ** $0.05<P<0.01$; *** $P<0.001$.
