# Supplementary material for: Characterization of Purple Acid Phosphatase Family and Functional Analysis of GmPAP7a/7b Involved in Extracellular ATP Utilization in Soybean
Source: Front Plant Sci. 2020 Jun 24;11:661. doi: 10.3389/fpls.2020.00661 (PMC7326820; doi:10.3389/fpls.2020.00661)
Supplement: TABLE S1 — Primers used for qRT-PCR or vector construction. [file Data_Sheet_2.PDF]

TABLE S1. Primers used for qRT-PCR and vector construction

Gene name	Forward primer (5'-3')	Reverse primer (5'-3')
GmPAP1a	TTTAGTGCTTCCACATGTCCA	GCTACTAGCTTTGGGTTTGAC
GmPAP18a	ACATGGACTAGTCAGTAAATGG	TCTAAAATCTCAATGTCAGTTAAGAATAT
GmPAP22a	ACGCTCAATCACAACAATACAC	TATCGTAAATTCGAGTGAAGCGC
GmPAP20a	ACCTCCTCGTACCGTTATCTG	TTTGTCCAAGATCTCCAACTACTG
GmPAP27c	ATGCTGTCTAACGACCAACTC	AAACTCCACATTCTCCACCTG
GmPAP27a	ATGAATCTGGTTCTGGTAGTGG	GTATCCTCACCCTTTGTGCC
GmPAP7d	CCTTGTGTTTGGTTGTTTCCTC	GGATCATCTATGCAGTGACCA
GmPAP7a	GCATGTTATTCCCTCTTATGTTCG	ACAATTCCCATCTGATTGGCG
GmPAP17d	TAGCAGAAGCAGCCAAATCC	GCAGAGACAGAAGTACCTAACC
GmPAP17a	AATGGAGTTGCGTTGCGTTG	GGAAACTGACATGGGTTGCG
GmPAP12a	AAGCACTGTCAAATACTGGG	TGGTGTCAAACTCCAAATTGTG
GmPAP12b	TCGGTAGATATGCCAATAGACAG	GGTGTCAAACTCCAAATTGTG
GmPAP27b	GCTAGGAACTAAGGGTGAGGA	ATAGTTTCGGTTAGAGTAGTTGGC
GmPAP17b	CCTTTCGTGCTTATTCCACTG	TGCAGCGTGATGTGAATGTG
GmPAP7e	AAGAGATGGCTTTGCCTGAG	CTAAATCCACATCCTTGAGTAGGT
GmPAP7c	TTCTCAAGGACGTGGATTTGG	CTGTCAAGGCTACTTATATGTTCC
GmPAP7b	GCATGGTATTCCCTGTTATGGT	AATTCCCATCTGATTGGCGA
GmPAP27d	GATGATATGTGCAGTTCAACGC	TTCTTCTGAAGCATCAAGAGGAG
GmPAP10a	GACCCTGGATCACGGGTTTT	CCCATGACACGATCACTGCT
GmPAP1b	GGGACGAAATAACTGTAACATGG	GCTTGTATTTGTACTCTTGGTTGG
GmPAP18b	TATGGATGGCTAAAGGAAGATCTG	TATGGACTGAACCACAAGGA
GmPAP15a	TCTCTACCTCCCATGACTCC	GCTTGGTTCCAATCCTTTGAG
GmPAP22b	CTTCCCTTACCTATTGACAAAGTC	CGAAATGTGCACCTGTTGAG
GmPAP15b	GGAGATGTTAGTTATGCCAACC	TCTACCCTCATACACTCTGCT
GmPAP10b	AATAAATACGCGAGTGTTGCGA	GCGTTATATGAACCTGCTGGG
GmPAP27e	ACTTGGAGGAATGTTGTGCCAT	TGCAGAAGCTGTCATTCTCAGG
GmPAP10c	TGTTTGAGCCTTGGTTTGTG	ATTTGTCATGTTGCTTGCCA
GmPAP26a	AAGGAGTGCAGCATATCAGC	GGCGTGTACTTCACAAATGG
GmPAP10d	GTGAACCAATCGATGCTAATAGG	ATAATGATTAGTGTGATTGTTGATGAAGA
GmPAP18c	GCCAGGTAGTTACTGTTGCTG	GAAATGTGTACCTGTTGAGGG
GmPAP26b	ATATACAAAGTGGAGCGCAGAC	AACTTCTCCCATGTAAGGCA
GmPAP15c	CTTCAATGGACATGTTCATGCG	CACCTCTAGAATGCCATGACC
GmPAP1c	TCATGGTTACAAGAACACAGGA	CCAAGTTACAGTCATTTCATCCC
GmPAP23	GAAAGTGTCCTTCTGCTGGT	AATTCACAACCTCAAGTATCCC
GmPAP22c	CATCGTTGGTGACTTGGGAC	TTGCCTTCGGTAACCATCCA
GmPAP20b	CCTCTTTCTCCTTCGTTCGTC	CTTGAGATATGTGTACCTGTTGAG
GmPAP17c	AGGAAATCAACTGCAAGATGG	TAGAAGTGGGCTATCTGTGCT
GmPAP9	ATTATTACAAGGTTGGAAATGATAACG	GAGGATCCACTTCATGGTTGATATG

TABLE S1 continued

Gene name	Forward primer (5'-3')	Reverse primer (5'-3')
GmEF1-α	TGCAAAGGAGGCTGCTAACT	CAGCATCACCGTTCTTCAAA
AtEF1-α	GTCGATTCTGGAAAGTCGACC	AATGTCAATGGTGATACCACGC
GmPAP7a-OX	GCGAGCTCGGTACCCGGGAATCTCTCTAT-CCCTCCCTCC	CTCTAGAGGATCCCCGGGTTATATCCAGGC-TGCTACGTTAC
GmPAP7b-OX	GCGAGCTCGGTACCCGGGGAGAACTTGC-AGCGCAGGTC	CTCTAGAGGATCCCCGGGTTATATCCAGGC-TGCTACGTTAC
GmPAP7a-GFP	CTCTAGCGCTACCGGTAATCTCTCTATCCC-TCCCTCC	CATGGTGGCGACCGGTAGTATCCAGGCTG-CTACGTTAC
GmPAP7b-GFP	CTCTAGCGCTACCGGTGAGAACTTGCAGC-GCAGGTC	CATGGTGGCGACCGGTAGTATCCAGGCTG-CTACGTTAC
GmPAP7a-GST	GGGGCCCCTGGGATCCAATCTCTCTATCC-CTCCCTCC	GGGAATTCGGGGATCCTTATATCCAGGCTG-CTACGTTAC
GmPAP7b-GST	GGGGCCCCTGGGATCCGAGAACTTGCAG-CGCAGGTC	GGGAATTCGGGGATCCTTATATCCAGGCTG-CTACGTTAC
GmPAP7a-GUS	CTATGACATGATTACGAATTCCTCCTAATT-TCTGACTTCAGTTACCG	GACTGACCTACCCGGGGATCCGGTTTAAT-TTTAAGGACCTGCAAG
GmPAP7b-GUS	CTATGACATGATTACGAATTCTCTGAATCA-CACCTTCACTCTTG	GACTGACCTACCCGGGGATCCGCTTACGC-TCTAGCGAGTAGAAAC

TABLE S2. Relative expression levels of *GmPAP* members

Gene name	2 d Leaf		16 d Leaf		2 d Root		16 d Root	
	+P	-P	+P	-P	+P	-P	+P	-P
GmPAP1a	0.46±0.01	0.42±0.04	0.60±0.08	3.50±0.10	0.54±0.07	0.59±0.08	0.41±0.13	4.35±0.52
GmPAP18a	0.89±0.18	0.88±0.26	1.59±0.18	1.38±0.24	0.67±0.04	0.86±0.05	2.54±0.32	3.01±0.40
GmPAP22a	1.01±0.14	0.95±0.07	0.33±0.11	0.89±0.18	0.91±0.11	0.74±0.10	0.20±0.18	0.21±0.09
GmPAP20a	0.98±0.18	1.01±0.22	0.34±0.14	0.39±0.10	0.96±0.18	1.87±0.08	0.09±0.04	0.63±0.18
GmPAP27a	0.40±0.02	0.36±0.03	0.88±0.10	2.73±0.12	0.66±0.01	1.24±0.08	1.26±0.06	2.36±0.13
GmPAP7d	0.97±0.11	0.66±0.05	0.11±0.07	0.06±0.02	0.97±0.11	0.66±0.05	0.11±0.07	0.06±0.02
GmPAP7a	0.16±0.02	0.16±0.02	0.09±0.06	7.61±0.99	0.28±0.01	0.46±0.03	0.53±0.08	9.40±0.13
GmPAP17d	1.00±0.02	0.94±0.04	0.20±0.01	1.64±0.07	0.37±0.04	0.75±0.10	0.11±0.01	13.17±1.23
GmPAP17a	0.79±0.11	0.62±0.13	1.58±0.26	1.06±0.11	0.56±0.09	0.63±0.06	1.94±0.30	3.74±0.16
GmPAP12a	0.15±0.05	0.13±0.03	2.91±0.55	13.15±1.07	0.54±0.11	0.79±0.08	1.10±0.29	5.40±0.31
GmPAP12b	0.03±0.02	0.06±0.03	0.20±0.08	10.51±0.26	0.42±0.05	0.44±0.06	0.39±0.12	12.13±0.21
GmPAP27b	1.17±0.04	1.24±0.05	0.03±0.01	0.25±0.02	0.46±0.03	0.64±0.07	2.27±0.19	5.96±0.39
GmPAP17b	0.83±0.08	0.93±0.15	1.88±0.19	1.30±0.14	0.65±0.07	0.75±0.08	1.86±0.15	4.06±0.15
GmPAP7e	0.87±0.08	0.70±0.06	0.27±0.11	0.17±0.06	0.63±0.02	1.66±0.05	1.23±0.19	1.03±0.07
GmPAP7c	nd	nd	nd	nd	0.79±0.03	0.66±0.05	1.42±0.24	1.29±0.12
GmPAP7b	0.12±0.03	0.19±0.01	0.19±0.05	6.40±0.44	0.22±0.05	0.21±0.04	0.72±0.09	8.65±0.36
GmPAP1b	0.62±0.04	0.73±0.09	0.20±0.02	1.53±0.12	0.81±0.06	1.18±0.07	0.84±0.08	3.58±0.20
GmPAP18b	1.28±0.03	1.18±0.05	0.10±0.02	0.24±0.01	1.04±0.02	0.97±0.08	0.63±0.04	1.23±0.11
GmPAP15a	0.34±0.06	0.45±0.04	0.93±0.10	4.31±0.24	1.09±0.04	1.15±0.08	0.51±0.09	0.80±0.17
GmPAP27d	1.01±0.06	0.77±0.03	0.13±0.08	0.16±0.08	0.62±0.04	0.87±0.05	0.98±0.12	4.30±0.09
GmPAP10a	0.19±0.09	0.31±0.02	0.2±0.02	0.23±0.02	nd	nd	nd	nd
GmPAP22b	0.22±0.07	0.14±0.09	0.41±0.10	7.25±0.69	0.55±0.03	1.08±0.02	0.62±0.06	2.47±0.02
GmPAP10b	0.78±0.03	0.72±0.02	0.29±0.01	3.31±0.05	0.66±0.01	0.86±0.04	0.86±0.06	4.55±0.33
GmPAP10c	0.97±0.10	0.68±0.04	1.37±0.13	0.80±0.28	0.77±0.03	0.90±0.06	2.15±0.17	4.33±0.46
GmPAP26a	1.02±0.03	1.05±0.03	0.14±0.01	0.22±0.01	0.61±0.08	0.63±0.11	1.75±0.12	3.55±0.13
GmPAP10d	1.31±0.18	0.96±0.04	0.33±0.07	0.23±0.06	1.28±0.06	0.100±0.03	1.52±0.08	1.13±0.26
GmPAP18c	0.66±0.01	0.49±0.07	1.27±0.18	1.53±0.10	1.42±0.12	1.91±0.05	2.04±0.24	2.30±0.20
GmPAP26b	1.14±0.03	1.11±0.02	0.31±0.01	0.65±0.01	0.97±0.03	1.10±0.06	0.78±0.04	1.15±0.06
GmPAP15c	1.54±0.21	0.93±0.29	0.19±0.02	0.04±0.02	0.62±0.01	0.72±0.07	0.79±0.06	0.58±017
GmPAP1c	0.17±0.02	0.18±0.01	0.32±0.01	0.77±0.02	0.84±0.04	1.08±0.07	0.68±0.07	1.61±0.09
GmPAP23	0.87±0.01	0.83±0.03	0.44±0.08	1.91±0.05	0.26±0.02	0.13±0.03	0.58±0.02	10.54±0.58
GmPAP22c	0.49±0.06	0.68±0.16	0.80±0.06	1.68±0.13	0.80±0.06	1.01±0.10	1.32±0.11	4.14±0.09
GmPAP20b	1.05±0.15	0.89±0.20	0.88±0.14	1.16±0.16	0.84±0.03	1.06±0.06	0.14±0.01	0.45±0.06
GmPAP17c	1.31±0.19	1.09±0.18	0.20±0.03	0.21±0.02	nd	nd	nd	nd
GmPAP9	0.50±0.03	0.60±0.07	1.76±0.06	3.95±0.26	0.76±0.03	0.94±0.04	1.00±0.08	2.16±0.06

Note: nd means that no expression was detected.
